# Association between Body Mass Index (BMI) and Dental Caries among 6–12-Year-Old School Children

**DOI:** 10.3390/children9050608

**Published:** 2022-04-25

**Authors:** Sunil Babu Kotha, Shayma Abdulaziz Terkawi, Sarah Ali Mubaraki, Abdulrahman Dahham Al Saffan, Sree Lalita Kotha, Sreekanth Kumar Mallineni

**Affiliations:** 1 Preventive Dentistry Department, Pediatric Dentistry Division, College of Dentistry, Riyadh Elm University, Riyadh 13244, Saudi Arabia; shema.a.terkawe@student.riyadh.edu.sa (S.A.T.); sarah.mubaraki@riyadh.edu.sa (S.A.M.); 2 Department of Pediatric and Preventive Dentistry, Sharad Pawar Dental College and Hospital, Datta Meghe Institute of Medical Sciences, Wardha 442004, Maharashtra, India; 3 Public Health Dentistry Division, Preventive Dentistry Department, College of Dentistry, Riyadh Elm University, Riyadh 13244, Saudi Arabia; a@riyadh.edu.sa; 4 Department of Basic Dental Sciences, College of Dentistry, Princess Nourah bint Abdulrahman University, Riyadh 11671, Saudi Arabia; sreelalitacelur@gmail.com; 5 Department of Preventive Science, College of Dentistry, Majmaah University, Almajmaah 11952, Saudi Arabia; 6 Center for Transdisciplinary Research (CFTR), Saveetha Dental College, Saveetha Institute of Medical and Technical Sciences, Saveetha University, Chennai 600077, Tamilnadu, India

**Keywords:** body mass index, children, dental caries, decayed

## Abstract

This study aimed to identify the association between BMI and dental caries in 6–12-year-old children. This cross-sectional study was carried out among 6–12-year-old school children and their parents. The data on Body Mass Index (BMI) and dental caries for study participants were included in the study. The association between BMI and dental caries was evaluated using SPSS software. The study comprises 400 school children (157 boys and 243 girls) aged an average of 8.9 years. The overall prevalence of dental caries was 84% in primary dentition and 75% in permanent dentition, with a mean DMFT and dmft (decayed, missing and filled teeth) of 2.85 and 5.48, respectively. There was a significant association witnessed between mothers’ education and BMI status. A significant association was also evident between decayed (d), missing (m) and filled (f) teeth and overall dmft with different BMI categories (*p* < 0.05). There was no significant association evident between DMFT and each category of BMI (*p* > 0.05). The dmft and DMFT within the four BMI categories by one-way ANOVA were highly significant (*p* < 0.001). Post hoc analysis helped us identify the relationship among the various categories of BMI with dental caries. There was a positive association evident between the BMI of the children and dental caries.

## 1. Introduction

Although it is internationally accepted that assessing height and weight is a reliable indicator of general health and wellbeing [1], it has been reported that abnormal weight leads to deprived impacts, including growth, health, quality of life and health problems in children [1,2,3,4]. Saudi Arabia has been dynamically Westernized in recent decades, and is now an expanding overweight population [5]. Increasing weight is now a genuine concern in Saudi Arabia, where 70 percent of society encounters this issue [6]. Dental caries are common in children, regardless of numerous preventive strategies like the utilization of fluoride as dentifrices, mouthwashes or fissure sealants, which may be due to inadequate plaque removal, and above all, the dietary habits of consuming more significant amounts of sugary and highly retentive food [2]. The prevalence of dental caries was significantly higher among Saudi Arabian school children; it was estimated to be around 80% for primary dentition, with an average dmft (decayed, missing and filled teeth) score of 5, and a DMFT score of 3.5 for permanent dentition [7]. 

A person’s weight in kilograms divided by their height in square meters is measured as body mass index (BMI). Globally, BMI and dental caries constitute fundamental health problems among children [3,4]. Parents and caretakers need to obtain sufficient knowledge through legitimate professional support to handle these problems [2]. Before finding out the common risk factor among them, it was essential to identify previous studies that have discussed the relationship between them. Many studies showed contradictory relationships. Various studies [2,8] reported that the results were not conclusive to prove the relationship between them, but suggested further research and other confounding variables that play a significant role in the association between dental caries and weight. Previously published studies established that dental caries and body mass index (BMI) are directly associated [2,9,10,11,12,13]. There are mixed opinions reported in the literature regarding BMI status and dental caries. Chen et al. [14] reported a positive association between overweight children with dental caries. The authors concluded that children in high-income countries are more prone to be overweight than those in low- and middle-income countries. A systematic review by authors from Saudi Arabia concluded that the BMI values would vary between countries [15]. Another systematic review done by Pasi et al. al [16] reported an inconsistent association between dental caries and BMI status. On the other hand, some research showed no association between BMI and dental caries [17,18,19]. Recently, a couple of authors reported from various parts of Saudi Arabia established an association between dental caries and dental caries [19,20,21,22,23]. Few researchers observed a positive relationship between dental caries and BMI [20,21], and some other studies found a negative association between BMI and dental caries [19,22], while a few other studies observed non-significant observations [6,17]. The majority of the studies focused on children from different parts of Saudi Arabia, while none of the researchers focused on Riyadh children of age 6–12 years. Therefore, the study aimed to identify the relationship between BMI and dental caries among school children aged 6–12-years-old.

## 2. Materials and Methods

The Riyadh Elm University scientific committee has approved the proposal under ethical approval of RC/IRB/2016/335. After the study was approved, permission was obtained from the deans of respective schools to conduct the survey. A cross-sectional study was conducted among primary school children aged 6–12 years old in the 2017–18 academic year. The G* Power Statistical Software (version 3.1.9.2) was used to calculate a minimum sample size of 400 participants. Simple random sampling was employed to select participants, and 20 schools were chosen randomly in Riyadh city. It was planned that approximately 20 children were to be randomly examined in each primary school between 6 and 12 years of age, based on inclusion and exclusion criteria, to achieve a sample of 400. A prior explanation of the purpose of the study to the parents and their children assured them of confidentiality, for which all the participants agreed to provide their personal information regarding the purpose and the procedures of our study. The inclusion criteria were Saudi natives, healthy children aged between 6–12 years and children whose parents signed the informed consent. Non-Saudi national children, those undergoing orthodontic treatment and those not medically fit were excluded from the analysis. The study was conducted in two phases. The brief questionnaire, which was about the age and gender of their child, the parent’s educational level and their financial status, was first filled out by the parent. 

The height and weight were recorded, followed by an oral examination of the child. The child was asked to stand straight after removing their footwear. The standing height was estimated to be the closest full centimeter, utilizing a measuring scale (Model: Seca 213 portable Stadiometer height-rod). The weight was noted using an electronic librated scale, and rounded off to the nearest 1 kg. BMI was computed by dividing the weight by kilograms and height squared in meters. BMI was categorized into underweight (BMI < 18.5), normal (BMI 18.5–24.9), overweight (BMI 25–30) and obese (BMI > 30) [24]. The children were seated on a comfortable chair under natural light using sterile portable equipment, including a mirror, explorer and cotton pellets. Dental caries was diagnosed according to World Health Organization oral health survey [25]. 

The collected data were tabulated in a Microsoft Excel sheet. The two examiners were selected to examine the children, and the method followed was to overcome the bias. The kappa statistics showed excellent (0.8 and 0.9) intra—(Examiner 1 and Examiner 2) and good (0.7) inter-examiner reliability. The relationship between the four BMI groups and the demographic factors (gender, mother’s and father’s education and family’s financial status), DMFT and dmft within caries-free and caries-active groups were evaluated and tested. The association of DMFT and dmft with BMI status was assessed using One-Way-Anova and post hoc analysis. Pearson’s correlation was utilized between dmft/(dmft + DMFT) and BMI status to determine the relationship between caries and weight status among the genders. A value of *p* < 0.05 was considered statistically significant, while *p* < 0.001 was considered highly effective.

## 3. Results

Overall, 400 children participated in the study of age 6–12 years. Among them, 60%, 243) of the subjects were girls, and 40% were boys (157). 34% (135) of the study population were underweight, while average weight, overweight and obese children were 26%, 22% and 19%, respectively. 30% of the girls were normal weight and 19% were obese, while 18% of boys were obese and 19% were normal weight. The relationship between the BMI categories and the demographic factors (gender, mother’s and father’s education and the family’s financial status) was summarized in Table 1.

The BMI of the subjects was given in four groups, where only a minor percent (26%) of the participants were in the normal range of 18.5–24.9 BMI. A significant association with *p* < 0.05 was found between the four categories of BMI about gender. The Chi-Square test did not reveal any significant relationship between the father’s education and socioeconomic condition and the children’s BMI status. However, the mother’s education was significantly associated with the BMI status of the child. The numbers and percentages of participants in the four categories of BMI in relation to the presence or absence of dmft are shown in Table 2. 

Table 2 also shows the individual categories of BMI concerning the presence or absence of dmft. The results show a highly significant (*p* < 0.001) relationship among the BMI categories for both decayed and missing teeth, particularly in the underweight, overweight and obese categories. Similar results are seen for filled teeth, which was significant (*p* < 0.05) among the BMI categories. The individual categories of BMI in relation to the presence or absence of DMFT are shown in Table 3. The results show that decayed teeth are more common in children in the underweight and overweight categories (54.5%) of BMI, compared to normal children (17.75%), although this was not statistically significant. The rate of missing permanent teeth is almost negligible, irrespective of any of the categories of BMI, and was also not significant (*p* > 0.05). DMFT scores were almost equally distributed, and were slightly more in underweight children (*p* > 0.05).

**Figure 1:**
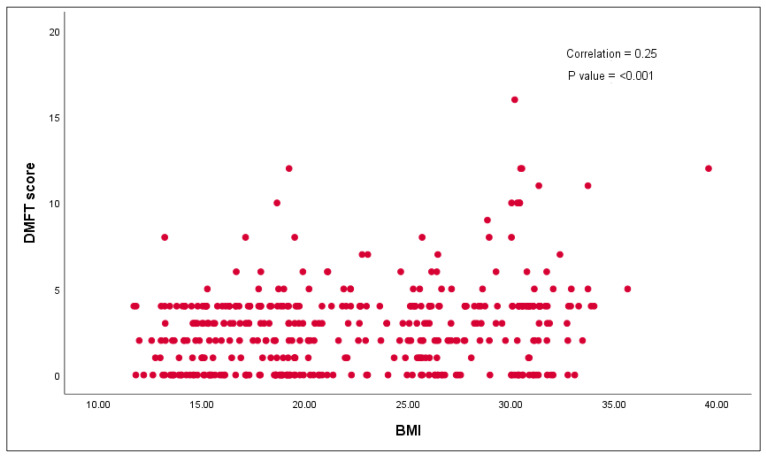
Association between overall DMFT scores and BMI scores.

**Figure 2:**
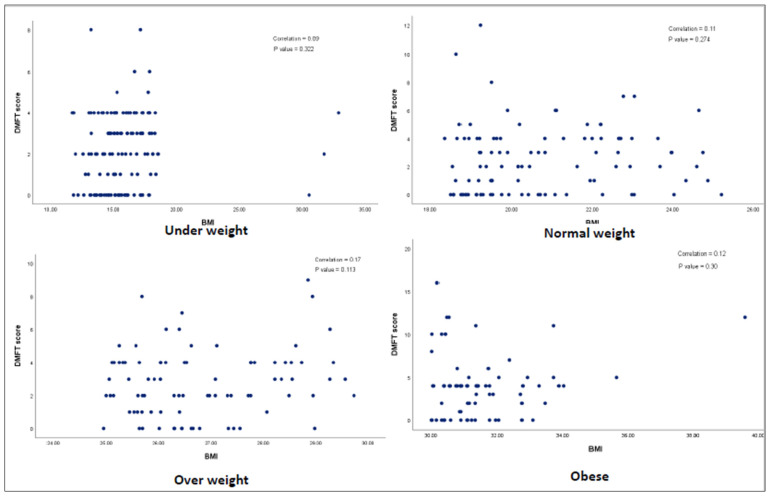
Association between DMFT score and BMI score among various BMI categories.

Figure 1 There is evidence to show that a positive association (r = 0.25) between overall scores of BMI and DMFT and statistical significance was evident (*p* < 0.05). Figure 2 explains the association based on gender and BMI status. There was no evidence of a statistically significant association between BMI status and DMFT among various BMI groups, including underweight, normal weight, overweight and obese children. All the comparisons are illustrated in Figure 2.

## 4. Discussion

Various studies [19,20,21] have been reported from Saudi Arabia regarding the relationship between dental caries and BMI. The Avon Longitudinal Study of Parents and Children (ALSPAC) [26], which considered 5000 children in their research from birth to 15 years, found that the maximum number of children changing from a healthy weight to overweight or becoming obese was much higher between 7 and 11 years than between 3 and 7 years or 11 and 15 years of age. Based on this study, the study sample used in the present study was aged 6–12 years. In the present study of the selected age group of 6–12 years, it was found that there is a female preference for increased BMI. The prevalence of increasing weight and dental caries in children is a concern in Saudi Arabia [27,28,29,30,31,32]. Alshihri et al. [33] reported that obesity and dental caries are multifactorial diseases, and the establishment of an association between these two diseases is very complex. Therefore, the objective of the present study was to identify the relationship between these two prevalent factors in school children, as the results of this research may help plan and promote healthcare at a very early age. The present study’s analysis revealed that increased weight and obesity conditions showed a positive association with prevalent dental caries, and the results were statistically significant. 

The current study results also showed that girls had a higher BMI than boys, which could be because females are more prone to increased weight and obesity due to anxiety, stress and depression due to changes in puberty, leading to increased levels of cortisol promoting weight gain [34]. Mothers with no school education resulted in an increased chance of obesity in their children [35,36,37]. In the present study, maternal education and employment were directly related to the child’s BMI (*p* < 0.05), unlike paternal education, which was not statistically significant. The truth behind the significance is that the mother spends comparatively more time with children than their fathers [37], which might explain why maternal education has a more significant effect than paternal education [31]. Mothers also have greater responsibility for checking on their children’s dietary habits than fathers [35]. The present study revealed that the relationship between socioeconomic status and obesity did not show any statistical significance. This interaction [38] is a complex relationship. In underdeveloped low-income countries, people with good financial conditions tend to have more obese children due to increased consumption of a high-calorie diet and less physical activity, unlike the children of developed countries where incomes are high. Individuals with good financial conditions are less obese as they are more conscious of their eating habits and physical health. This may be due to many community and preventive programs in developed countries that influence people with high socioeconomic status, making them more conscious about their health [39,40,41,42]. Being a developed country with high salary slabs, Saudi Arabia showed similar results with reduced obesity (though not statistically significant) in high socioeconomic groups [43].

The overall prevalence of dental caries in primary dentition was 84%. In permanent dentition, it was 75% among the total number of children examined, with a mean DMFT of 2.85 and DMFT of 5.48. The present study showed that around 40.25% of participants were overweight or obese, which was higher than the estimated trends in obesity and overweight prevalence in Saudi Arabia [44], which was estimated to be 30.1% (27.3%–33.0%). Saudi Arabia has experienced tremendous monetary and social changes over the past few decades [45,46]. Fast urbanization, overwhelming dependence on vehicles, multiplied satellite television and telecommunication automation and lessened occupational work demands have added to the enormous lifestyle changes, including expanded, inactive practices and diminished physical activity [45,47]. This habit of sedentary life among children in Saudi Arabia has led to children becoming addicted to video games for approximately 4 h a day on average [48]. These factors increase weight, and therefore BMI. This can be aggravated by the predominant intake of a high-calorie diet, including foods such as fries, cakes, ice creams and chocolates [49]. The increased prevalence of obesity is consistent with other previous studies of the Middle East [19,20,21,22].

The authors hypothesized a positive association between BMI and dental caries in 6–12-year-old children, and found a significant relationship between them. The present study findings revealed that BMI is one of the predicting factors for dental caries, and vice versa. As BMI is a sign of health, it is a valuable instrument to determine a person’s health. For the vast majority of the population, a normal BMI range is 18.5 to 24.9. The BMI scale is one of the nutritional screening or assessment tools used for screening malnutrition. Underweight individuals have a BMI of 18.5 or lower, which is interchangeable with malnutrition [49]. Prior studies [50,51] have shown that dental caries in primary dentition have an association with malnutrition during childhood; however, malnutrition in permanent dentition has not been reported in the published literature among Arabian children. Hypoplasia of enamel [50], hypofunction of salivary glands [50,52] and altered composition of saliva [50] are the effects of malnutrition, resulting in increased dental caries, but these individual effects are beyond our study. 

Children with low BMI show that increased dental caries can happen bi-directionally. One is that malnutrition or decreased BMI results in increased dental caries because of the causes mentioned above (hypoplasia, hyposalivation and altered composition of saliva). In a reversed way, the other reason is that the pain due to the advancement of dental caries leads to reduced food intake, resulting in malnutrition and reduced BMI [53,54,55]. Furthermore, severe dental caries associated with iron deficiency anemia posed indigent development and reduced BMI [54]. Johansson et al. [52] showed that many of the protective constituents (total protein, immunoglobulins, lactoperoxidase and lysozyme) are depressed in malnutrition conditions, resulting in an increased number of caries. It has been reported that buffer capacity and lower salivary pH promote carious activity in cystic fibrosis. However, this study was performed in adults, and our findings were not comparable [56]. The present study findings also exhibited that the dental carious lesions in underweight school children were comparatively high. 

Previously published studies [16,57] reported that increased weight and obesity are more likely caused by potential risk factors relating to a high frequency of sugared foods and between-meal snacks, resulting in increased dental caries. However, the dietary habits of the participants are beyond this study. The authors opine that this is a prime reason for increased BMI, particularly among schoolchildren in urban areas of Saudi Arabia [19,26,58]. Ruottinen et al. [59] examined children from infancy to 10 years, and concluded that constant intake of high sucrose increases weight and dental caries. Comparably, several studies associated BMI with dental caries, which gave very contradictory results with direct [2,9,10,11,12], indirect [13] or no association being evident among them [18,19]. The present study directly associated increased weight and obesity with dental caries. This could be due to reduced buffering mechanisms [60,61] and increased streptococcal count [62]. 

The sample size in the study was 400, although the sample was only from Riyadh school children. A study with a larger sample size would be imperative to generalize the findings. Selecting a particular population group (school children) may make it difficult to generalize this data to other geographic areas. Other dependent factors like the role of constituents of saliva, dietary habits and physical activity status should have been included to reach a tapering conclusion. Still, the uniqueness of the present study is that we investigated ages between 6 and 12 years and focused on both genders, unlike the previously published studies that were focused only on males or females individually. In the present study, however, the authors observed a positive association between dental caries and BMI status among Arabian children living in Riyadh. The authors in the present study used the same BMI categories for both genders based on the findings utilized in prior studies from Saudi Arabia [26]. The use of different BMI categories for boys and girls might provide a more realistic insight into the association with dental caries. The majority of the studies from Saudi Arabia focus on dental caries and BMI status [20,21,22,23]; none of the studies discussed parental education and BMI status in 6–12-year-old children. This study establishes the association between parental education and BMI status, a key finding in the present study. There was a positive association between dental caries and BNI status in the present study, and these findings agree with earlier studies [21,22]. Abu El Qomsan et al. [21] analyzed 386 children of 6–12 years of age from AlKurj City, Saudi Arabia, while Ashour et al. [22] involved 275 special care female participants aged 6–17 years residing in Makkah City, Saudi Arabia. Nevertheless, in the present study, 400 children aged 6–12 years participated from Riyadh city, Saudi Arabia. It is imperative to distinguish the hazard in early childhood by taking necessary measurements and planning a strategy to improve weight-related issues (either malnutrition or obesity) and dental caries. For this, this is not just the responsibility of pediatricians or pediatric dentists; it should be a multidisciplinary approach for ‘healthy weight’ and ‘healthy teeth’.

## 5. Conclusions

This study identified greater DMFT in obese and overweight children. Among Arabian children, girls were observed with a higher BMI compared to boys. However, the mother’s education was significantly associated with the BMI status of the child. There was no significant relationship between the father’s education or socioeconomic condition and the children’s BMI status. 

## Figures and Tables

**Table 1:** Demographic characteristics concerning BMI status categories (Chi-Square test).

Details		Underweight	Normal Weight	Overweight	Obese	*p*-Value
Gender	Male	64 (16%)	30 (7.5%)	35 (8.75%)	28 (7%)	0.03 *
	Female	71 (17.75%)	74 (18.5%)	51 (12.75%)	47 (11.75%)	
Mother’s education	Below high school	23 (5.75%)	5 (1.25%)	11 (2.75%)	12 (3%)	0.03 *
	High school	29 (7.25%)	24 (6%)	28 (7%)	22 (5.5%)	
	Graduation and above	83 (20.75%)	75 (18.75%)	47 (11.75%)	41 (10.25%)	
Father’s Education	Below high school	24 (6%)	9 (2.25%)	20 (5%)	20 (5%)	0.06 ^NS^
	High school	40 (10%)	33 (8.25%)	24 (6%)	22 (5.5%)	
	Graduation and above	71 (17.75%)	62 (15.5%)	42 (10.5%)	33 (8.25%)	
Financial status	<5000 riyals	42 (10.5%)	22 (5.5%)	29 (7.25%)	30 (7.5%)	0.19 ^NS^
	5000–10,000 riyals	44 (11%)	39 (9.75%)	25 (6.25%)	24 (6%)	
	>10,000 riyals	49 (12.25%)	43 (10.75%)	32 (8%)	21 (5.25%)	

* = Significant; NS = Non-significant.

**Table 2:** Relationship between dental caries (dmft) among the primary teeth with BMI status categories.

Primary Teeth	Underweight	Underweight	Normal Weight	Overweight	*p*-Value	*p*-Value
decayed (d)	Present	119 (29.75%)	49 (12.25%)	69 (17.25%)	62 (15.5%)	<0.001 **
	Absent	16 (4%)	55 (13.75%)	17 (4.25%)	13 (3.25%)	
missing (m)	Present	58 (14.5%)	34 (8.5%)	53 (13.25%)	51 (12.75%)	<0.001 **
	Absent	77 (19.25%)	70 (17.5%)	33 (8.25%)	24 (6%)	
filled (f)	Present	51 (12.75%)	20 (5%)	33 (8.25%)	23 (5.75%)	0.009 **
	Absent	84 (21%)	84 (21%)	53 (13.25%)	52 (13%)	
dmft	Present	130 (32.5%)	67 (16.75%)	80 (20%)	67 (16.75%)	<0.001 **
	Absent	5 (1.25%)	37 (9.25%)	6 (1.5%)	8 (2%)	

**-Highly Significant.

**Table 3:** Association of DMFT and dmft with BMI status along with post hoc. (one-way ANOVA with post hoc).

Index	Underweight	Normal Weight	Overweight	Obese	*p*-Value	Post hoc
DMFT	2.24 (1.785)	2.41 (2.371)	2.77 (2.033)	4.00 (3.553)	<0.001 *	4 > 3 = 2 = 1
dmft	4.56 (2.387)	2.38 (2.486)	6.49 (2.913)	8.49 (4.225)	<0.001 *	4 > 3 > 1 > 2

D = decayed; M = missed; F = filled; T = Teeth; dmft = primary teeth; DMFT = permanent teeth, *-Significant.

## Data Availability

The data that support the findings of this study are available from the corresponding author upon reasonable request.

## References

[1] Heinrich-Weltzien R., Monse B., Benzian H., Heinrich J., Kromeyer-Hauschild K. (2013). Association of dental caries and weight status in 6- to 7-year-old Filipino children. Clin. Oral Investig..

[2] De Jong-Lenters M., van Dommelen P., Schuller A.A., Verrips E.H. (2015). Body mass index and dental caries in children aged 5 to 8 years are attending a dental pediatric referral practice in the Netherlands. BMC Res. Notes.

[3] Bjorge T., Engeland A., Tverdal A., Smith G.D. (2008). Body mass index in adolescence in relation to cause-specific mortality: A follow-up of 230,000 Norwegian adolescents. Am. J. Epidemiol..

[4] Whitlock G., Lewington S., Sherliker P., Clarke R., Emberson J., Halsey J., Qizilbash N., Collins R., Peto R., Prospective Studies Collaboration (2009). Body-mass index and cause-specific mortality in 900,000 adults: Collaborative analyses of 57 prospective studies. Lancet.

[5] DeNicola E., Aburizaiza O.S., Siddique A., Khwaja H., Carpenter D.O. (2015). Obesity and public health in the Kingdom of Saudi Arabia. Rev. Environ. Health.

[6] Farsi D.J., Elkhodary H.M., Merdad L.A., Farsi N.M., Alaki S.M., Alamoudi N.M., Bakhaidar H.A., Alolayyan M.A. (2016). Prevalence of obesity in elementary school children and its association with dental caries. Saudi Med. J..

[7] Al-Agili D.E. (2013). A systematic review of population-based dental caries studies among children in Saudi Arabia. Saudi Dent. J..

[8] Hayden C., Bowler J.O., Chambers S., Freeman R., Humphris G., Richards D., Cecil J.E. (2013). Obesity and dental caries in children: A systematic review and meta-analysis. Community Dent. Oral Epidemiol..

[9] Gerdin E.W., Angbratt M., Aronsson K., Eriksson E., Johansson I. (2008). Dental caries and body mass index by socio-economic status in Swedish children. Community Dent. Oral Epidemiol..

[10] Willershausen B., Moschos D., Azrak B., Blettner M. (2007). Correlation between oral health and body mass index (BMI) in 2071 primary school pupils. Eur. J. Med. Res..

[11] Powell J.C., Koroluk L.D., Phillips C.L., Roberts M.W. (2013). Relationship between adjusted body mass index percentile and decayed, missing and filled primary teeth. J. Dent. Child.

[12] Yao Y., Ren X., Song X., He L., Jin Y., Chen Y., Lu W., Guo D., Ding L., Tang H. (2014). The relationship between dental caries and obesity among primary school children aged 5–14 years. Nutr. Hosp..

[13] Bafti L.S., Hashemipour M.A., Poureslami H., Hoseinian Z. (2015). Relationship between body mass index and tooth decay in a population of 3–6-year-old children in Iran. Int. J. Dent..

[14] Chen D., Zhi Q., Zhou Y., Tao Y., Wu L., Lin H. (2018). Association between Dental Caries and BMI in Children: A Systematic Review and Meta-Analysis. Caries Res..

[15] Alshehri Y., Park J.S., Kruger E., Tennant M. (2020). Association between body mass index and dental caries in the Kingdom of Saudi Arabia: Systematic review. Saudi Dent. J..

[16] Paisi M., Kay E., Bennett C., Kaimi I., Witton R., Nelder R., Lapthorne D. (2019). Body mass index and dental caries in young people: A systematic review. BMC Pediatr..

[17] Ashi H., Campus G., Klingberg G., Forslund H.B., Lingström P. (2019). Childhood obesity in relation to sweet taste perception and dental caries—A cross-sectional multicenter study. Food Nutr. Res..

[18] Sadeghi M., Alizadeh F. (2007). Association between dental caries and body mass index-for-age among 6–11-year-old children in Isfahan in 2007. J. Dent. Res..

[19] Quadri M.F., Hakami B.M., Hezam A.A., Hakami R.Y., Saadi F.A., Ageeli L.M., Alsagoor W.H., Faqeeh M.A., Dhae M.A. (2017). Relation between dental caries and body mass index-for-age among schoolchildren of Jazan city, Kingdom of Saudi Arabia. J. Contemp. Dent. Pract..

[20] Abu El Qomsan M.A., Alasqah M.N., Alqahtani F.A., Alobaydaa M.A., Alharbi M.M., Kola Z. (2017). Intricate Evaluation of Association between Dental Caries and Obesity among the Children in Al-Kharj City (Saudi Arabia). J. Contemp. Dent. Pract..

[21] Ashour N.A., Ashour A.A., Basha S. (2018). Association between body mass index and dental caries among special care female children in Makkah City. Ann. Saudi Med..

[22] Alghamdi A.A., Almahdy A. (2017). Association Between Dental Caries and Body Mass Index in Schoolchildren Aged Between 14 and 16 Years in Riyadh, Saudi Arabia. J. Clin. Med. Res..

[23] Swaminathan K., Anandan V., SelvaKumar H., Thomas E. (2019). Correlation Between Body Mass Index and Dental Caries Among Three- to 12-Year-Old Schoolchildren in India: A Cross-Sectional Study. Cureus.

[24] Alswat K., Mohamed W.S., Wahab M.A., Aboelil A.A. (2016). The association between body mass index and dental caries: Cross-sectional study. J. Clin. Med. Res..

[25] World Health Organization (1997). Oral Health Surveys—Basic Methods.

[26] Reilly J.J., Dorosty A.R., Emmett P.M. (2000). Identification of the obese child: Adequacy of the body mass index for clinical practice and epidemiology. Int. J. Obes. Relat. Metab. Disord..

[27] Farsi D.J., Elkhodary H.M. (2021). The prevalence of overweight/obesity in high school adolescents in Jeddah and the association of obesity association with dental caries. Ann. Saudi Med..

[28] Alsaif A.A., Alkhadra T.A., AlJameel A.H. (2021). Oral health-related quality of life among groups of foundling and delinquent children in comparison with mainstream children. J. Popul. Ther. Clin. Pharmacol..

[29] Abdellatif H., Hebbal M.I. (2020). Dental Caries and Its Association with Body Mass Index among School Children of Riyadh, Saudi Arabia. J. Pharm. Bioallied Sci..

[30] Al-Dossary S.S., Sarkis P.E., Hassan A., Ezz El Regal M., Fouda A.E. (2010). Obesity in Saudi children: A dangerous reality. East. Mediterr. Health J..

[31] Al Shehri A., Al Fattani A., Al Alwan I. (2013). Obesity among Saudi children. Saudi J. Obes..

[32] Alshihri A.A., Rogers H.J., Alqahtani M.A., Aldossary M.S. (2019). Association between Dental Caries and Obesity in Children and Young People: A Narrative Review. Int. J. Dent..

[33] Ashour A.A., Basha S., Tenan E., Basalem A., Al Qahatani A. (2019). Association between obesity/overweight and dental caries in psychiatric patients. Ann. Saudi Med..

[34] Block J.P., He Y., Zaslavsky A.M., Ding L., Ayanian J.Z. (2009). Psychosocial stress and change in weight among US adults. Am. J. Epidemiol..

[35] Lamerz A., Kuepper-Nybelen J., Wehle C., Bruning N., Trost-Brinkhues G., Brenner H., Hebebrand J., Herpertz-Dahlmann B. (2005). Social class, parental education and obesity prevalence in a study of six-year-old children in Germany. Int. J. Obes. Relat. Metab. Disord..

[36] Muthuri S.K., Onywera V.O., Tremblay M.S., Broyles S.T., Chaput J.P., Fogelholm M., Hu G., Kuriyan R., Kurpad A., Lambert E.V. (2016). Relationships between Parental Education and Overweight with Childhood Overweight and Physical Activity in 9–11 Year Old Children: Results from a 12-Country Study. PLoS ONE.

[37] Fuemmeler B.F., Lovelady C.A., Zucker N.L., Østbye T. (2013). Parental obesity moderates the relationship between childhood appetitive traits and weight. Obesity.

[38] Hsin A., Felfe C. (2014). When does time matter? maternal employment, children’s time with parents and child development. Demography.

[39] Patrick D.L., Lee R.S., Nucci M., Grembowski D., Jolles C.Z., Milgrom P. (2006). Reducing oral health disparities: A focus on social and cultural determinants. BMC Oral Health.

[40] Alyousef A.M., Almehrej B.A., Alshahrani M.A., Almutairi K.M., Alqasir M.A., Alassaf A., Almulhim B., Alghamdi S., Mallineni S.K. (2021). Arabian Parents’ Knowledge, Attitude and Practice towards their Children’s Oral Health and Early Childhood Caries Resided in Riyadh Province: An Online-Based Cross-Sectional Survey. Ann. Med. Health Sci. Res..

[41] Kumar V., Ankola A., Sankeshwari R., Jalihal S., Atre S., Mallineni S.K. (2021). Determination of the oral health status and behaviors, treatment needs, and guardians’ perception of oral health among preschool children attending Integrated Child Developmental Scheme Anganwadi centers of Belagavi, South India: A cross-sectional study. J. Clin. Transl. Res..

[42] Khan S.Q., Khan N.B., Arrejaie A.S. (2013). Dental caries. A meta analysis on a Saudi population. Saudi Med. J..

[43] Martinez R. (2015). Prevalence of Overweight and Obesity Visualization. http://publichealthintelligence.org/content/prevalence-overweight-and-obesity-worldwide.

[44] Kandelman D., Arpin S., Baez R.J., Baehni P.C., Petersen P.E. (2012). Oral health care systems in developing and developed countries. Periodontology.

[45] AlBlehed A.K., AlThumairy A.F., AlTurayri W.S., Alassaf A., Almulhim B., Alghamdi S., Almalki A., Mallineni S.K. (2021). Assessment of Knowledge, Attitude and Practices Regarding Oral Hygiene among the Parents of Pre-School Children: A Cross-Sectional Study: A Cross-Sectional Study. Ann. Med. Health Sci. Res..

[46] Al-Hazzaa H.M., Al-Nakeeb Y., Duncan M.J., Al-Sobayel H.I., Abahussain N.A., Musaiger A.O., Lyons M., Collins P., Nevill A. (2013). A cross-cultural comparison of health behaviors between Saudi and British adolescents living in urban areas: Gender by country analyses. Int. J. Environ. Res. Public Health.

[47] Andersen R.E., Crespo C.J., Bartlett S.J., Cheskin L.J., Pratt M. (1998). Relationship of physical activity and television watching with body weight and level of fatness among children: Results from the Third National Health and Nutrition Examination Survey. JAMA.

[48] Bhayat A., Ahmad M.S., Fadel H.T. (2016). Association between body mass index, diet and dental caries in Grade 6 boys in Medina, Saudi Arabia. East. Mediterr. Health J..

[49] Pinson R.D. (2011). Body Mass Index and Malnutrition: Interrelated Comorbidities. http://www.hcpro.com/HOM-271737-5728/Body-Mass-Index-and-malnutrition-Interrelated-comorbidities.html.

[50] Psoter W.J., Katz R.V., Reid B.C. (2005). Malnutrition and dental caries: A review of the literature. Caries Res..

[51] Xavier A., Bastos R.D.S., Arakawa A.M., De Lourdes Caldana M., De Magalhães Bastos J.R. (2013). Correlation between dental caries and nutritional status: Preschool children in a Brazilian municipality. Rev. Odontol. UNESP.

[52] Johansson I., Ericson T., Bowen W., Cole M. (1985). The effect of malnutrition on caries development and saliva compostion in the rat. J. Dent. Res..

[53] Mitrakul K. (2016). Assessing associations between caries prevalence and body mass index and nutrition data among children aged in 6–12 years. Southeast Asian J. Trop. Med. Public Health.

[54] Anil S., Anand P.S. (2017). Early childhood caries: Prevalence, risk factors and prevention. Front. Pediatr..

[55] Alkarimi H.A., Watt R.G., Pikhart H., Sheiham A., Tsakos G. (2014). Dental caries and growth in school-age children. Pediatrics.

[56] Hooley M., Skouteris H., Boganin C., Satur J., Kilpatrick N. (2012). Body mass index and dental caries in children and adolescents: A systematic review of literature published 2004 to 2011. Syst. Rev..

[57] Hildebrandt T., Zawilska A., Trzcionka A., Tanasiewicz M., Mazurek H., Świętochowska E. (2020). Estimation of Proinflammatory Factors in the Saliva of Adult Patients with Cystic Fibrosis and Dental Caries. Medicina.

[58] Alm A., Fåhraeus C., Wendt L.K., Koch G., Andersson-Gäre B., Birkhed D. (2008). Body adiposity status in teenagers and snacking habits in early childhood in relation to approximal caries at 15 years of age. Int. J. Paediatr. Dent..

[59] Ruottinen S., Karjalainen S., Pienihakkinen K., Lagstrom H., Niinikoski H., Salminen M., Rönnemaa T., Simell O. (2004). Sucrose intake since infancy and dental health in 10-year-old children. Caries Res..

[60] Ain T.S., Sultan S., Gowhar O., Ravishankar T.L., Kumar S. (2016). Obesity and salivary parameters (flow rate, buffer capacity and salivary ph) in children of morabad India. Int. J. Sci. Study.

[61] Choromanska K., Choromanska B., Dabrowska E., Baczek W., Mysliwiec P., Dadan J., Zalewska A. (2015). Saliva of obese patients—Is it different?. Postepy Hig. Med. Dosw..

[62] Barkeling B., Linne Y., Lindroos A.K., Birkhed D., Rooth P., Rossner S. (2002). Intake of sweet foods and counts of cariogenic microorganisms in relation to body mass index and psychometric variables in women. Int. J. Obes. Relat. Metab. Disord..

